# Eating disorders, bipolar disorders and other mood disorders: complex and under-researched relationships

**DOI:** 10.1186/s40337-019-0262-2

**Published:** 2019-09-12

**Authors:** Claire McAulay, Phillipa Hay, Jonathan Mond, Stephen Touyz

**Affiliations:** 1 Clinical Psychology Unit, Brain & Mind Centre, University of Sydney, Sydney, Australia; 2 Translational Health Research Institute, School of Medicine, Western Sydney University, Sydney, Australia; 3 Centre for Rural Health, College of Health and Medicine, University of Tasmania, Sydney, Australia; 4 Inside Out Institute, Charles Perkins Centre, Sydney Medical School, University of Sydney, Sydney, Australia

**Keywords:** Mood, Bulimia nervosa, Anorexia nervosa, Binge eating disorder, Mania, Hypomania, Depression.

## Background

Eating disorders are understood to no longer be the remit of lean adolescent girls alone [1], whatever pop culture offerings such as Netflix’s *To The Bone* might continue to present. Rather, people with eating disorders are much more diverse, often undiagnosed and often suffering in silence. One patient population with a unique but poorly understood vulnerability to eating disorders, as well as obesity and poor physical health, is people with bipolar disorder.

Bipolar disorder is a complex, serious mental illness that confers significant functional impairment, primarily due to time spent in manic, depressed, or mixed state episodes, difficulty eliminating subthreshold depressive symptoms, and the financial, social and emotional sequelae of illness episodes. One significant contributor to disability is physical ill health, with the average life expectancy of someone with bipolar disorder notably reduced due to cardiometabolic illnesses like heart disease and type two diabetes mellitus. Furthermore, poorer physical health is associated with poorer mental health in this group, such as higher risk of depressive relapse [2, 3].

The past two decades has seen increased focus on preserving and improving the physical health of people with bipolar disorder, who are often prescribed long-term medication known to be conducive to weight gain [4]. Trials of a number of interventions have attempted to implement lifestyle interventions to promote healthy eating, physical activity, and social connectedness, in order to prevent or reduce the adverse impact of obesity and improve physical health and psycho-social functioning [5]. These have been of limited long-term effectiveness, though early intervention post-first episode psychosis appears to be more promising [6].

The role of disordered eating in contributing to physical and mental health outcomes among individuals with bipolar disorder has rarely been considered, despite the fact that approximately one in three people with bipolar disorder also meet criteria for binge eating disorder, bulimia nervosa, or variants of these disorders [7, 8]. Alongside studies questioning the impact of medication on weight gain in bipolar disorder [9], identifying the role of weight-cycling in higher cardiovascular risk [10], and showing that obesity risk pre-dates medication [11], are reports of a link between eating disorders and high Body Mass Index (BMI, kg/m^2^) [12] and evidence that baseline binge eating predicts medication-associated weight gain [13].

With these recent developments in mind, the authors were curious about what novel interventions for eating disorders and weight disorder in bipolar disorder exist, and conducted a systematic review about their effectiveness.^1^ In this search no treatments targeting eating disorders specifically were found to exist [14], despite the movement of this area into the spotlight over 10 years ago [15]. Most treatments of people with a high BMI in bipolar disorder neglect psychological factors with the exception of [16], and people with bipolar disorder are often though not always; see [17] excluded from eating disorder treatment trials e.g. [18, 19]. Hence, the effectiveness of Cognitive Behaviour Therapy (CBT) or other common treatments for eating disorders have not been evaluated among individuals with this comorbidity.

One reason for the lack of treatment protocols is our poor understanding of the detail of eating disorders in bipolar disorder. This includes: whether eating disorders in bipolar disorder resemble eating disorders in those without bipolar disorder; what modifiable factors predict eating disorder risk; which temperamental factors or psychological mechanisms, such as impulsivity and emotion regulation, may be at play, and to what extent,; when eating disorder and bipolar disorder features first present and subsequently interact across the lifespan; and whether antipsychotic use remains relevant to obesity risk to when controlling for other factors. While one theory argues that impulsivity is probably the shared factor between mania and binge eating [20], other studies have suggested that higher impulsivity in this group is not unique enough to this subgroup, being elevated in bipolar-only patients, and is likely subsumed by other variables such as eating-disordered cognitions [21].

To answer these questions, the authors (CM, JM and ST) have undertaken a series of studies, in Australia and the Netherlands, addressing these and other aspects of the association between bipolar disorder and eating disorders, using a comprehensive assessment of eating disorder features so as to avoid the loss of information inherent in categorical assessment. Preliminary findings have been instructive with our team able to isolate weight and shape overvaluation, dietary restriction and binge eating as particularly common, and unique contributors to poorer quality of life, among people with bipolar disorder [22]. Furthermore, data from both countries has highlighted that emotion regulation difficulties, but not impulsivity, are independently associated with eating disorder symptoms after controlling for other relevant factors.

While this editorial is the beginning, rather than conclusion, of a conversation about eating disorders in bipolar disorder, it is hoped that by sparking interest in the area, novel treatments can be more widely considered for this challenging presentation. As McElroy and colleagues have argued [23], more consideration should be paid to novel approaches to this comorbidity, such as utilising psychotherapy approaches typically used in both bipolar disorder and eating disorder-only populations. We argue that there is no obvious reason that enhanced CBT or schema-based approaches for eating disorders would not be effective for eating disorder and comorbid bipolar disorder patients. Similarly, Interpersonal and Social Rhythms Therapy, which emphasises healthy routines around sleep, activity and diet, has been proposed as a feasible approach for this group, and some of its principles may be easily adapted for more specific eating disorder treatment targets [24]. Targeting emotion regulation and eating-disordered cognitions, relating to binge eating in particular, may offer benefits for quality of life, psychological distress and physical health in people with bipolar disorder– once we accept the challenge of launching a systematic investigation.

We hope that this research will complement existing studies into the association between mood disorders and eating disorders. As well as finding elevated rates of mood disorders in eating disorders [25], and binge eating in mood disorders [26], these comorbid subgroups may be more complex [27], but conversely, more responsive to CBT treatments [28, 29]. Furthermore, depressive symptoms may be more relevant to quality of life than the severity of eating disorder features [30]. While possible pathways of action include the relationship of mood and eating with perfectionism [31] and substance use disorders [32], the lack of intervention trials targeting comorbid eating disorders and mood disorders [33] makes it difficult to definitively identify why and how these features interact. However, many of the aforementioned authors advocate an approach that considers subtypes or phenotypes of eating disorders and mood disorders, rather than considering these to be homogenous groups. This could subsequently help identify clinical processes and inform hypotheses in comorbid eating disorders and bipolar disorder, including what factors may predict or hinder treatment response.

To further research in this important area more broadly, the Journal of Eating Disorders is initiating a Special Issue on mood disorders and eating disorders. We encourage submissions that consider the interplay between multiple psychiatric illnesses in these categories, measure burden in these populations, and identify specific features that are responsible for poorer outcomes if found to exist. Papers exploring novel approaches to management of emotion dysregulation as well as specific mood disorders as experienced by people with eating disorders will be particularly welcome.

## Data Availability

Data sharing not applicable to this article as no datasets were generated or analysed during the current study.

## Abbreviations

BMI: Body Mass Index
CBT: Cognitive Behavioural Therapy

## Footnotes

1 Unpublished research. McAulay C, Touyz S, Malhi G, Mond J, & Outhred T. When Targeting Physical Health in Bipolar Disorder, Do Researchers Consider Psychological Factors? A Systematic Review. Conference Presentation, Society for Mental Health Research; 2016; Sydney.
